# *Streptomyces* Strains Induce Resistance to *Fusarium oxysporum* f. sp. *lycopersici* Race 3 in Tomato Through Different Molecular Mechanisms

**DOI:** 10.3389/fmicb.2019.01505

**Published:** 2019-07-03

**Authors:** Sakineh Abbasi, Naser Safaie, Akram Sadeghi, Masoud Shamsbakhsh

**Affiliations:** ^1^Department of Plant Pathology, Faculty of Agriculture, Tarbiat Modares University, Tehran, Iran; ^2^Department of Microbial Biotechnology, Agricultural Biotechnology Research Institute of Iran (ABRII), Agricultural Research, Education and Extension Organization (AREEO), Karaj, Iran

**Keywords:** Fusarium wilt, induced systemic resistance, *Streptomyces*, tomato growth promoting, siderophore production

## Abstract

Plant growth promoting rhizobacteria (PGPR) are potential natural alternatives to chemical fungicides in greenhouse production via inducing plant immune system against biotic stresses. In this research, 126 *Streptomyces* isolates were recovered from rhizosphere soils of 13 different commercial vegetable greenhouses in Iran. *Streptomyces* isolates were screened for *in vitro* Plant growth promoting (PGP) traits and ability to antagonize *Fusarium oxysporum* f. sp. *lycopersici* race 3 (*FOL*), the causal agent of Fusarium wilt of tomato (FWT). Six isolates with the highest antagonistic activity and at least three PGP traits were selected and compared with chemical fungicide Carbendazim^®^ in a greenhouse experiment. All bacterial treatments mitigated FWT disease symptoms like chlorosis, stunting and wilting at the same level or better than Carbendazim^®^. Strains IC10 and Y28 increased shoot length and shoot fresh and dry weight compared to not inoculated control plants. Phenotypic characterization and 16S rRNA gene sequencing showed, strains IC10 and Y28 were closely related to *S. enissocaesilis* and *S. rochei*, respectively. The ability of the superior biocontrol strains to induce antioxidant enzymes activity and systemic resistance (ISR) was investigated. Increased activity of catalase (CAT) in plant treated with both strains as well as an increase in peroxidase (POX) activity in plants treated with Y28 pointed to a strain specific-induced systemic resistance (ss-ISR) in tomato against *FOL*. The differential induced expression of *WRKY70* and *ERF1* (two transcription factors involved in plant defense) and *LOX* and *TPX* by the analyzed *Streptomyces* strains, especially after inoculation with *FOL*, suggests that ss-ISR is triggered at the molecular level.

## Introduction

In recent decades, overuse of chemical fertilizers and fungicides to proliferate and protect greenhouse vegetables has been a threat to human safety. Using biological agents is the best alternative for chemical treatments (Pilkington et al., 2010). Soil-borne fungus *Fusarium oxysporum* Schlecht. f. sp. *lycopersici* (Sacc.) Snyder and Hansen (*FOL*) causing Fusarium wilt of tomato reduces yield in greenhouses (Fravel et al., 2003; McGovern, 2015). Invading through the vascular tissue and soil- borne feature of the pathogen make it difficult to control this disease. Besides, new races of the mentioned pathogen (e.g., race 3) have emerged that can overcome host resistance (Reis et al., 2005). The symptoms of Fusarium wilt disease caused by race 3 are yellowing the lower leaves, vascular necrosis, epinasty, defoliation, plant stunting and ultimately plant death (Sanchez-Pena et al., 2010). Biological control of this pathogen has been the subject of many studies (Ben Abdallah et al., 2016). Plant growth promoting rhizobacteria (PGPR) colonize rhizosphere or plant root and improve plant health and growth. Some of the most important plant growth promoting (PGP) activity include diazotrophic nitrogen fixation, siderophore production, solubilization of mineral phosphates and production of hormones such as indole-3-acetic acid (IAA) (Sadeghi et al., 2012; Dahal et al., 2017; Gouda et al., 2018). Plant defense strategies including physical barriers, numerous secondary metabolites and antimicrobial agents, which the effective, aggressive pathogens have to overcome them (reviewed by Bruce and Pickett, 2007). Some plant defense strategies are constitutive while others are inducible and only launch in response to a stimulating pathogen and/or beneficial microbes (Pieterse et al., 2012). Plant defense strategies keep damage of specific pests below an economic threshold; however, maintain the beneficial organisms involved in integrated pest management (IPM) (Chellemi et al., 2016). IPM considers available pest control techniques that prevent the development of pest populations and keep pesticides to levels that are economically reasonable and reduces risks to human health and the environment. Developing sustainable biocontrol measures for managing Fusarium wilt disease requires a comprehensive understanding of the molecular basis of plant–pathogen–biocontrol interactions. Hormones, PR proteins, terpenoid synthases, polyketide terpene synthases, peroxidases, lignin synthases, transcription factors, calcium signal transducers, and UDP-glucosyltransferases and ubiquitin-protein ligases are components of the plant defense-related genes (Wang et al., 2015). Generally, the plant responses to microbes are regulated through signaling pathways including salicylic acid (SA), jasmonic acid (JA), and ethylene (ET). Jasmonic acid pathway is required for defense against necrotrophic pathogens and chewing insects, while SA pathway is involved in a wide range of plant defense responses, which ends to systemic acquired resistance (SAR) and occurs following the exposure to many biotrophic and some necrotrophic pathogens. Induced systemic resistance (ISR) is also an activated resistance process elicited by contacting with non-pathogenic microorganisms. This procedure is independent of SA and is synchronized by JA and ET (Walters and Heil, 2007). Activated induced resistance (via ISR or SAR) is a broad-spectrum and long-term resistance, which usually suppresses a disease up to 20–85% (Walters et al., 2013). Thus, inducing plants by direct interaction with rhizobacteria prior to pathogen infection, so-called priming, decreases disease severity (Beckers and Conrath, 2007). *Streptomyces* species, are Gram-positive filamentous bacteria, reported as PGP and biocontrol agent of *Alternaria alternata* (Verma et al., 2011), *Rhizoctonia solani* (Goudjal et al., 2014), *F. oxysporum* (Kim et al., 2011), *Phytophthora drechsleri* (Sadeghi et al., 2017), and *Verticillium dahliae* (Cao et al., 2016). Little is known about bio-suppression of tomato wilt by *Streptomyces* in greenhouse conditions.

The aims of this study were to (1) isolate and characterize *Streptomyces* from vegetable greenhouse soils (2) detect PGP characteristics and antagonistic activity of isolates against *FOL* race (3) evaluate Fusarium wilt biocontrol in tomato by superior isolates in greenhouse condition (4) establish induced systemic resistance (ISR) of tomato through inducing antioxidant enzymes and defense-related genes by inoculation of plants with biocontrol *Streptomyces.*

## Materials and Methods

### Microorganisms

Rhizosphere soil samples (500 g soil with pH = 6.2–7 and EC < 2.5 dS/m) were collected from 18 cucumber and tomato commercial greenhouses in Yazd, Isfahan and Kerman provinces of Iran in 2016. There were no symptoms of wilt and damping off diseases developing in these greenhouses. The samples were placed in plastic bags, taken to the laboratory and then air-dried for 7 days. For isolation of *Streptomyces*, 2 g rhizosphere soil was suspended in 100 mL of sterile saline solution (0.9% NaCl) and shaken for 30 min. Two dilutions (1:100 and 1:1000) were prepared using sterile saline solutions in a final volume of 1 mL. An aliquot of 0.1 mL of each dilution was spread on 1.8% water agar supplemented with 300 mM NaCl. The plates were incubated at 29°C for 7 days. Representative colonies were selected and streaked on plates of “International Streptomyces Project media 2” (ISP2) medium (Kampfer et al., 1991) containing 10 g/L malt extract, 4 g/L yeast extract, 4 g/L glucose, and 18 g/L agar, adjusted to pH 7.2. The plates were incubated at 29°C for 7 days. Then morphologically (color, size, and shape) distinct colonies were stored in 30% glycerol solution at −70°C (Table 1).

**Table 1:** PGP properties and enzyme activity of selected isolates.

Isolate	Host crop	Location	PGP properties				Hydrolytic enzymes activity		
			Growth on N free medium	Inorganic P solubilization	Siderophore production	IAA production	Cellulase	Protease	Chitinase
IC6	Cucumber	Isfahan	1	0.1 ± 0.1	0.3 ± 0.1	9.2 ± 0.0	1.0 ± 0.0	0.3 ± 0.0	1.0 ± 0.0
IC10	Cucumber	Isfahan	1	0.2 ± 0.0	0.4 ± 0.0	27.1 ± 0.1	0.0 ± 0.0	1.0 ± 0.0	0.9 ± 0.1
IC13	Cucumber	Isfahan	1	0.3 ± 0.0	0.4 ± 0.0	32.3 ± 2.6	1.0 ± 0.0	0.6 ± 0.0	0.9 ± 0.1
IS8	Cucumber	Isfahan	1	1.9 ± 0.1	0.4 ± 0.0	21.9 ± 0.9	0.0 ± 0.0	0.4 ± 0.0	1.0 ± 0.0
SS12	Cucumber	Isfahan	1	0.1 ± 0.0	0.3 ± 0.0	24.1 ± 1.9	1.0 ± 0.0	0.5 ± 0.0	0.8 ± 0.1
CU122	Cucumber	Isfahan	1	0.2 ± 0.0	0.1 ± 0.0	20.5 ± 0.7	0.0 ± 0.0	0.0 ± 0.0	0.0 ± 0.0
IC15	Cucumber	Isfahan	1	0.3 ± 0.0	0.3 ± 0.0	9.6 ± 1.2	0.0 ± 0.0	0.3 ± 0.0	0.9 ± 0.0
SS14	Cucumber	Isfahan	1	0.0 ± 0.0	0.3 ± 0.0	24.5 ± 0.7	1.0 ± 0.0	0.5 ± 0.0	0.0 ± 0.0
IT20	Tomato	Isfahan	1	0.2 ± 0.0	0.4 ± 0.0	24.0 ± 1.3	1.0 ± 0.0	0.5 ± 0.0	0.0 ± 0.0
IT8	Tomato	Isfahan	1	0.0 ± 0.0	0.4 ± 0.0	25.8 ± 0.5	1.0 ± 0.0	0.2 ± 0.0	0.0 ± 0.0
IT25	Tomato	Isfahan	1	0.2 ± 0.0	0.5 ± 0.0	25.8 ± 1.8	0.0 ± 0.0	0.3 ± 0.0	0.8 ± 0.2
Y7	Cucumber	Yazd	1	0.5 ± 0.1	0.0 ± 0.0	7.7 ± 0.9	0.0 ± 0.0	0.0 ± 0.0	0.0 ± 0.0
Y18	Cucumber	Yazd	1	0.1 ± 0.0	0.0 ± 0.0	12.0 ± 0.6	1.0 ± 0.0	0.9 ± 0.1	0.8 ± 0.2
Y27	Tomato	Yazd	1	0.0 ± 0.0	0.0 ± 0.0	30.8 ± 1.1	1.3 ± 0.4	0.3 ± 0.0	0.0 ± 0.0
TO612	Tomato	Yazd	1	0.0 ± 0.0	0.5 ± 0.0	24.7 ± 0.3	1.0 ± 0.0	0.0 ± 0.0	0.0 ± 0.0
Y17	Tomato	Yazd	1	0.0 ± 0.0	0.4 ± 0.0	13.7 ± 2.8	1.0 ± 0.0	0.4 ± 0.0	0.6 ± 0.3
Y28	Tomato	Yazd	1	0.4 ± 0.0	0.3 ± 0.0	16.8 ± 2.1	0.0 ± 0.0	0.0 ± 0.0	0.9 ± 0.0
Y281	Tomato	Yazd	1	0.3 ± 0.0	0.3 ± 0.0	10.0 ± 0.0	0.0 ± 0.0	0.0 ± 0.0	0.0 ± 0.0
Y16	Tomato	Yazd	1	0.0 ± 0.0	0.4 ± 0.1	11.4 ± 0.5	1.4 ± 0.4	0.0 ± 0.0	0.0 ± 0.0
Y33	Tomato	Yazd	1	0.2 ± 0.0	0.0 ± 0.0	9.3 ± 0.2	1.0 ± 0.0	0.3 ± 0.0	0.8 ± 0.2
Y34	Tomato	Yazd	1	0.3 ± 0.1	0.3 ± 0.0	11.8 ± 0.9	1.2 ± 0.2	0.2 ± 0.0	0.7 ± 0.3
KH12	Cucumber	Kerman	1	0.0 ± 0.0	0.1 ± 0.0	7.5 ± 0.7	0.2 ± 0.1	0.3 ± 0.1	0.0 ± 0.0
K40	Cucumber	Kerman	1	0.0 ± 0.0	0.0 ± 0.0	27.5 ± 2.2	0.2 ± 0.0	0.4 ± 0.0	1.0 ± 0.0
K43	Cucumber	Kerman	1	0.0 ± 0.0	0.0 ± 0.0	22.5 ± 3.5	1.0 ± 0.0	0.0 ± 0.0	0.0 ± 0.0

*The means halo zone diameter/ colony diameter of three replications ± standard deviation is represented. 1, growth; 0, no growth. The data indicated for IAA production was in μg/mL.*

The fungal pathogen was isolated from tomato plants displaying disease symptoms and pathogenicity test was conducted using root-dipping inoculation method on tomato seedlings. The fungus was re-isolated from the vascular tissue of a symptomatic plant. Fungal DNA extraction was carried out by the methods described by Zhang et al. (2010), Salehi et al. (2018, 2019). Polymerase chain reaction (PCR) was performed with universal pair primers (ITS4-ITS5). Race determination of fungus was done using four specific primer pairs designed by Hirano and Arie (2006) based on the sequence of the exo-polygalacturonase gene (Table 2). ITS4-ITS5 nucleotide sequence obtained by DNA amplification of fungal pathogen was deposited in NCBI GenBank with accession number MG670445. Blast analysis showed that this pathogen had 99 % similarity to *F. oxysporum*. The result of the race determination using four specific primer pairs showed that the fungal isolate belongs to *F. oxysporum* f.sp. *lycopersici* race 3 (Supplementary Figure S1).

**Table 2:** The list of primers used in the q-RT PCR in this study.

Gene Name	Amplicon size (bp)	Sequence
*UniF*	670	F-5′ATCATCTTGTGCCAACTTCAG3′ R-5′GTTTGTGATCTTTGAGTTGCCA3′
*Sp13*	445	F-5′GTCAGTCCATTGGCTCTCTC3′ R-5′TCCTTGACACCATCACAGAG3′
*Sp23*	518	F-5′CCTCTTGTCTTTGTCTCACGA3′ R-5′GCAACAGGTCGTGGGGAAAA3′
*Sprl*	947	F-5′GATGGTGGAACGGTATGACC3′ R-5CCATCACACAAGAACACAGGA3′
*UDP-G*	122	F-5′GATGAACGCCACCTTCTTAG3′ R-5′CTCCTTCCATAACAATCCTCAC3′
*WRKY70*	131	F-5′TGGTAAAGCATAGTGACTCAAC3′ R-5′AGAGGGAGAAGAAGGCATAA3′
*PAL1*	148	F-5′CATTGTACAGGTTGGTGAGAG3′ R-5′CATCTCTTGAGACACTCCA3′
*LOX E*	104	F-5′CTTCGGATACCCTTTACCT3′ R-5′GATCTCACCCAACTTCTTTC3′
*TPX*	133	F-5′AGCATTGACAACACGTACC3′ R-5′AGCACTCCCTGTCTTAACT3′
*PR1*	136	F-5′GGTAACTGGAGAGGACAA3′ R-5′GACAATCGATCACTTTATTC3′
*ERF1*	126	F-5′AGACTTGGGAGTTGAATTA3′ R-5′TACATTGCGATCTTGATTA3′
*TUB*	146	F-5′CAAGAACTCGTCCTACTTTG3′ R-5′GCTCACTCACCCTTCTAA3′

### Growth on Nitrogen-Free Medium

Screening of the free-living (non-symbiotic) diazotroph isolates was carried out according to the procedure described by Xu and Zheng (1986). Each colony was spot-inoculated on nitrogen-free agar medium incubated at 29°C for 14 days, and then growth or lack of growth was compared to ISP2 medium.

### Inorganic Phosphate Solubilization

Pikovskaya’s medium (PVK) was used to measure calcium phosphate [Ca_3_ (PO_4_)_2_]-solubilizing activity. Sterilized PVK medium with pH 7.2 was poured into petri plates. After solidification of the medium, bacterial isolates were spot-inoculated onto the center of the plates and incubated at 29°C for 7 days. Solubilization index was evaluated according to the ratio of the clear zone diameter to colony diameter (Soltani et al., 2010).

### Siderophore Production

Siderophore production was evaluated as described by Alexander and Zuberer (1991) on Chrome Azurol agar (CAS) medium. *Streptomyces* isolates were spot-inoculated onto the center of the plate and incubated at 29°C for 7 days. The formation of orange halo around the colonies were considered as siderophore-producing isolates. After 3 days, the ratio of the halo zone diameter to colony diameter was calculated as siderophore production.

### IAA Production

To determine amounts of IAA produced by each isolate, 1 mL of bacterial culture in ISP2 broth was inoculated in Tryptic Soy Broth supplemented with 150 mg/L L-tryptophan. Approximately 1 mL of culture solution was centrifuged at 12000 rpm for 5 min, and 1mL of the supernatant was mixed with 2 mL of Salkowski’s reagent (150 mL concentrated H_2_SO_4_, 250 mL distilled water, 7.5 mL 0.5 M FeCl_3_.6H_2_O) and incubated for 20 min in darkness at room temperature (de Oliveira-Longatti et al., 2014). IAA production was qualitatively assayed as pink color development and quantified by measurement of absorbance at 530 nm using a spectrophotometer infinit M200 (Tecan, Switzerland). The calibration plot was constructed using dilutions of a standard IAA (Fluka, Switzerland) solution and the uninoculated medium with the reagent as a standard curve (0, 5, 10, 20, 50, and 100 μg/mL). The quantity of IAA in the culture was expressed as μg/mL.

### Antagonistic Effect of PGPRs

Bacterial suspension of candidate PGPR isolates (Table 1) (20 μL of the 10^8^ cfu/mL sterile saline solution) was inoculated at 29°C linearly at the two opposite sides (1 cm from the plate edge) of potato dextrose agar (PDA) plates for 48 h. Then, one fungal plug (0.5 cm diameter) was inoculated at the center of PDA plate (Kunova et al., 2016) and then incubated for 4 days. The growth inhibition percentage was calculated using the formula (a − b)/a × 100, where “a” is the fungal growth radius of a control culture (in cm) and “b” is the distance of the pathogen growth in the direction of bacteria (in cm).

### Chitinase Activity

Chitinase production was determined according to the method of Hsu and Lockwood (1975). *Streptomyces* isolates were grown on chitin agar containing 0.4% colloidal chitin and 1.5% agar adjusted to pH 7.2. Colloidal chitin was prepared according to Berger and Reynolds (1958). Plates were incubated for 5 days at 29°C. The ability of chitinase production was shown by a clear halo around the colonies. The ratio of the clear zone diameter to colony diameter was calculated as chitinase activity.

### Cellulase Activity

Carboxymethyl cellulase (CMCase) activity was determined by Mandels-Reese medium with carboxymethyl cellulose (CMC) as sole carbon source (Majidi et al., 2011). The bacteria were grown on CMC agar containing 0.4 g/L KH_2_PO_4_, 0.02 g/L CaCl_2_, 0.02 g/L NaCl, 0.02 g/L FeSO_4_ 7H_2_O, 2.5 g/L CMC, and 15.0 g/L agar. The pH was adjusted to 7.2 with 1 M NaOH. The CMC agar plates were incubated at 29°C for 7 days to allow the secretion of cellulase. To visualize the hydrolysis zone, agar medium was flooded with an aqueous solution of Congo red (1 mg/mL) for 20 min. Congo red solution was then poured off, and the plates were further treated by flooding with 1 M NaCl for 15 min. To indicate cellulase activity, the diameter of the clear zone around each colony was measured. The ratio of the clear zone diameter to colony diameter was calculated as cellulase activity.

### Protease Activity

Extracellular protease activity of *Streptomyces* isolates was assayed by a modification method of Mehrotra et al. (1999). Each bacterial isolate was streaked on skim milk agar containing 15 g/L skim milk powder, 0.5 g/L yeast extract and 10 g/L agar. After incubation at 29°C for 48 h, the ratio of the clear zone around bacterial colony to colony diameter was measured as protease activity.

### Phenotypic and Molecular Characterization of the Selected Isolates

The potent PGP and antagonist isolates were further characterized by differential morphological traits on ISP2, ISP3, and ISP4 media (Shirling and Gottlieb, 1966), melanin formation (Suter, 1978), growth in high temperatures (37°C and 42°C), and growth on medium supplemented with 6, 10, and 12% NaCl (Kutzner, 1981).

Molecular characterization of the selected isolates was done using PCR amplification of 16S rRNA gene sequence. DNA was extracted according to the method described by Tripathi and Rawal (1998). PCR amplification was performed using the primers 27F: 5′-AGAGTTTGATCCTGGCTCAG-3′ and 1525R: 5′-AAAGGAGGTGATCCAGCC-3′ as described by Chun and Goodfellow (1995). Almost-complete 16S rRNA gene sequences (1400 nt) were deposited in the GenBank database under the accession numbers of MG722685 (strain IT25), MG786938 (strain TO612), MG786894 (strain Y17), MG786896 (strain Y28), MG654776 (strain IC10), and MG676358 (strain IC13). The sequences were aligned manually with corresponding sequences of available *Streptomyces* species deposited in the GenBank, EMBL and DDBJ databases using BLAST search tool (Altschul et al., 1997). Phylogenetic tree was constructed using the MEGA 6.0 software package (Tamura et al., 2013) based on neighbor-joining method. Bootstrap analysis was used to evaluate the stability of relationships based on 1000 resampling. Strains IC10 and Y28 were preserved and deposited in the Agricultural Biotechnology Research Institute of Iran Culture collection (ABRIICC) under accession numbers of ABRIICC 20108 and ABRIICC 20114, respectively.

### Greenhouse Experiments

Tomato (*Solanum lycopersicum* L.) cv. Rio Grande susceptible to *FOL* races 2 and 3 (Barker et al., 2005), was used in greenhouse experiments. Seeds were surface sterilized with 1% sodium hypochlorite (NaOCl) and incubated in a growth chamber at 25°C for 7 days. Germinated seedlings were placed into 84-cell plug tray (50 × 30 × 5 cm deep) filled with sterilized soil and peat moss (1:2), with one seedling occupying each cell. Seedlings were watered every day with tap water and kept at 27°C and 16 h brightness/8 h darkness. After 21 days, the seedlings were transferred to pots (15 × 20 cm) filled with a sterile mixture of field soil and peat moss (2:1). For bacterial treatments, *Streptomyces* cell and spore were centrifuged at 8000 rpm for 15 min and then the pellet re-suspended in 10 mL sterile saline solution. Bacterial suspension was added to autoclaved sand and final cfu/g sand adjusted to 10^6^. Seven gram of sand containing bacteria was added to the surface of each cultivated pot immediately after transferring of plant to the pot. Sterilized sand was used as a negative control. The pots were watered every 2 days. For *FOL* inoculation, 10 days after bacterial treats, tomato seedlings were uprooted and inoculated with conidial suspension for 30 min (root-dip method). Seedlings submerged in sterile distilled water (mock inoculation) were used for bacterial treatments and negative control. To prepare *FOL* suspension (pathogen inoculum), fungi was cultivated on PDA for 10 days. Conidia were harvested by scraping in sterile water (10 mL/plate) and final conidia/mL adjusted to 5 × 10^7^ using hemocytometer. The treatments were negative control plants (mock inoculation), positive control (*FOL* inoculated), Carbendazim^®^ (soil drenched with fungicide in a concentration of 1.5 g/L) and six *Streptomyces* treatments inoculated or non-inoculated with *FOL*. The plants were harvested 60 days after transferring to the pots. The growth parameters comprising of shoot length, fresh and dry weight of shoot and root were measured and recorded. Further, disease severity (DS) was assessed on a scale from 0 to 5: 1 = symptoms free = 0%; 2 = slight chlorosis, stunting, or wilting = 25%; 3 = moderate chlorosis, stunting, or wilting = 50%; 4 = severe chlorosis, stunting, or wilting = 75%; 5 = death = 100% (Marlatt et al., 1996). Percentage of control value was calculated using the formula (DC − DT)/DC × 100, where “DC” is disease index of inoculated control (*FOL*) and “DT” is disease index of inoculated treatment (%) (Song et al., 2004).

A greenhouse experiment was conducted separately for the next two tests. The procedure of this experiment was similar to the first one, but the experiment duration was shorter and the plants were harvested 14 days after transfer to the pots, also 1 cm of the root end of the seedlings was cut before *FOL*/mock inoculation. Foliar was sprayed with plant hormones (1 mM SA or 10 mM MeJA) 48 h before *FOL* inoculation. The treatments were negative control plants (mock inoculation), positive control (*FOL* inoculated), Carbendazim^®^ (soil drenched with fungicide in a concentration of 1.5 g/L) and two *Streptomyces* treatments (strains IC10 and Y28) inoculated or non-inoculated with *FOL*. Plants leaves were harvested 2 days after *FOL* inoculation, frozen in liquid nitrogen and kept at −70°C for the following analysis.

### Antioxidant Enzymes Activity

Frozen leaf samples (100 mg fresh weight) with 10 mg polyvinyl pyrrolydone (PVP) was transferred to 1.5 mL tube and homogenized in 1 mL Na-Pi buffer (1 mM, pH 7). The homogenate was centrifuged at 15000 × g for 15 min. All operations were performed at 4°C. The supernatant was used as a crude enzyme extract. The activities of antioxidant enzymes including peroxidase (POX: EC 1.11.1.7) and catalase (CAT: EC 1.11.3.6) were measured in a reaction containing 250 μL 0.1 M phosphate buffer (pH 7.0), 250 μL 10 mM guaiacol, 30 μL H_2_O_2_, and 40 μL crude enzyme extract (Chance and Maehly, 1955). The enzyme activity (U/mL) was measured spectrometrically (Cary 300, United States) by monitoring of the degrading H_2_O_2_ by the increase in the absorbance at 470 nm and the decrease in the absorbance at 290 nm over 3 min.

### Quantitative Real-Time PCR Analysis of the Defense-Related Genes

Total RNA was extracted from shoots using RNeasy plant mini kit (QIAGEN). cDNA was synthesized using 1 μg of each RNA sample after treating with RNase-free DNase I (Invitrogen) using iScript cDNA synthesis kit (BioRad) according to manual description. Quantitative PCR was performed in a 25 μL reaction containing 1 μL of template cDNA, 0.5 μL of 10 pM of each forward and reverse specific primer (Table 2) and iQ SYBR Green Supermix kit (BioRad) on BioRad multicolor real-time PCR detection system. The PCR profile included an initial denaturation step at 95°C for 3 min, followed by 40 cycles of denaturation (95°C/30 s), primer annealing (60°C/30 s) and primer elongation (72°C/30 s), by a final elongation step (72°C/ 5 min) and recording melting curves. Results were expressed as the normalized ratio of mRNA level of target gene to internal control tubulin gene (*TUB*). Changes was estimated by using the Relative Expression Software Tool (REST 2009) (Pfaffl et al., 2002).

### Statistical Analysis

Statistical analysis was performed using analysis of variance (ANOVA) by Microsoft Excel (Microsoft Corporation, United States) and SPSS version 16.0 (SPSS Inc. Chicago, IL, United States). All data shown are average value of three (*in vitro* experiments) biological replicates ± SE. The greenhouse experiments were carried out in randomized blocks design with four blocks and there were four biological replications for each treatment. The significance differences of the treatments were evaluated using multivariate generalized linear model (GLM) with Duncan multiple range test *post hoc* analysis at level of *P* < 0.05. The relationship between *in vitro* and *in vivo* data was studied using bivariate Pearson test at *P* ≤ 0.01.

## Results

### Isolation and Selection of *Streptomyces* Bacteria

**FIGURE 1:**
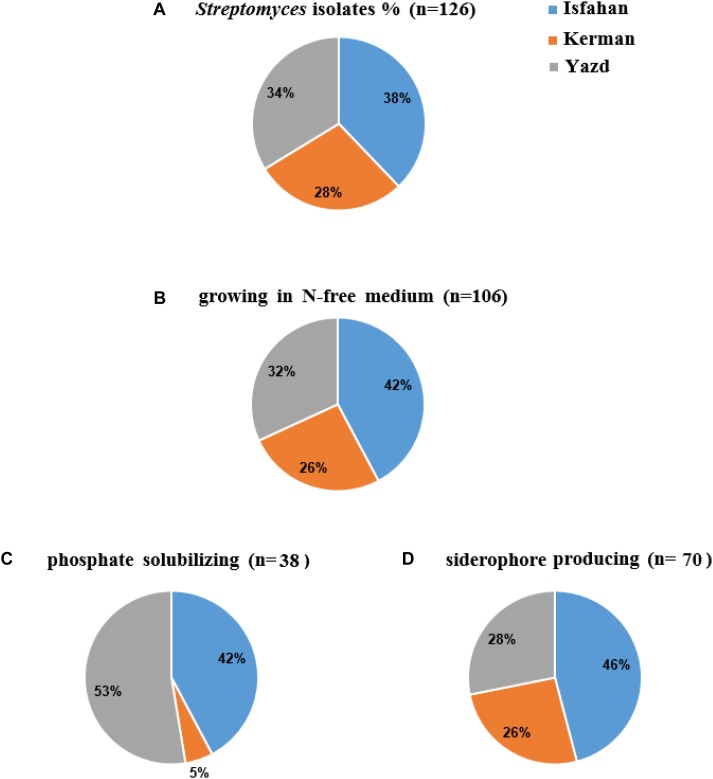
The percent (%) of colonies isolated from Isfahan, Yazd, and Kerman provinces: colonies **(A)** with *Streptomyces* appearance **(B)** growing in N-free medium, **(C)** phosphate solubilizing, and **(D)** siderophore producing.

A total of 126 colonies displaying *Streptomyces* appearance including compact colored heaped and wrinkled, waxy, chalky, powdery or velvety colony, were isolated from tomato and cucumber rhizosphere soil collected from Isfahan, Yazd and Kerman (Figure 1A). Totally, 106 isolates (84%) were able to grow on solid nitrogen-free medium (Figure 1B) of which thirty-eight isolates were able to solubilize inorganic phosphate. Three isolates comprising of IS8, Y7, and Y28 with the ratio 1.9, 0.5, and 0.4 had the most potential to solubilize tricalcium phosphate, respectively (Figure 1C). Further, sixty-six percent of 106 isolates were able to produce siderophore (Figure 1D). The maximum orange halo due to iron chelation was recorded for isolate TO612 after 7 days of incubation. All 106 isolates produced IAA in a range of 7.0–40.9 μg/mL of which twenty percent produced IAA more than 27 μg/mL. Thirty-two isolates from 106 showed proteolytic enzyme activity. The greatest halo zone/colony diameter ratio for protease activity was 1.0 and recorded for isolate IC10 (Table 1). Forty-four isolates were found to produce cellulose. The greatest halo zone/colony diameter ratio for cellulase activity was 1.4 which recorded for Y16. Furthermore, biosynthesis of the chitinolytic enzyme was detectable for 22 isolates. Isolates IC13, K40, SS12, IC6, and Y17 were able to produce all three examined hydrolytic enzymes. Overall, 24 isolates containing at least three PGP traits and hydrolytic enzymes activity were selected for evaluation in the following experiments as shown in Table 1.

### Growth Inhibition of the Selected Isolates Toward *FOL*

Six isolates (25% of the selected isolates) showed more than 30% inhibitory effect against *FOL* in dual culture test. The highest percentages of growth inhibition were 69, 49, 48, 42, 39, and 38, which were recorded for IC10, IT25, Y17, TO612, Y28, and IC13, respectively (Table 3). The biocontrol potential of these isolates (superior PGP antagonistic isolates) was evaluated in a greenhouse experiment.

**Table 3:** *In vitro* antagonistic activity against *Fusarium oxysporum* f.sp. *lycopersici* by selected isolates.

Isolates	Growth inhibition (%)	Isolates	Growth inhibition (%)	Isolates	Growth inhibition (%)
IC6	4.0 ± 0.1^j∗^	IT25	49.1 ± 0.8^b^	IC34	1.7 ± 1.5^j^
IC10	68.5 ± 1.0^a^	Y7	0.6 ± 1.1^j^	KH12	30.0 ± 0.0^d^
IC13	38.2 ± 0.9^c^	Y18	1.3 ± 1.1^j^	K40	4.0 ± 0.0^j^
IS8	10.6 ± 1.1^h^	Y27	0.6 ± 1.1^j^	K43	10.0 ± 2.0^h^
SS12	0.6 ± 1.1^j^	TO612	41.5 ± 1.8^c^		
CU122	0.0 ± 0.0^k^	Y17	47.5 ± 2.56^b^		
IC15	17.9 ± 1.4^g^	Y28	38.7 ± 1.1^c^		
SS14	1.0 ± 1.1^j^	Y281	2.3 ± 2.5^j^		
IT20	19.3 ± 0.8^f,g^	Y16	2.6 ± 2.5^j^		
IT8	9.6 ± 0.4^h^	Y33	1.6 ± 2.8^j^		

*Values are the means (averaged from three replicates) ± SE. ^∗^Same letters within each column represent non-significant difference according to Duncan’s Multiple Range Test (P < 0.05).*

### Phenotypic and Genotypic Characterization of the Superior Isolates

**FIGURE 2:**
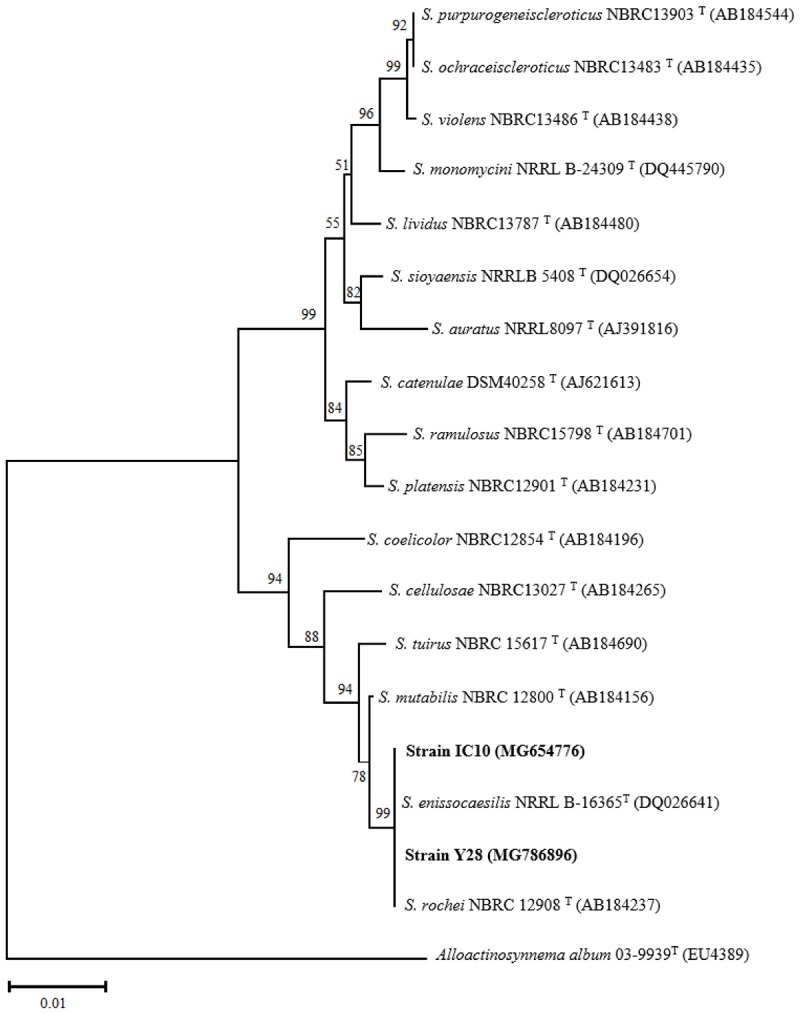
Phylogenetic tree based on almost complete 16S rRNA gene sequences of *S. enissocaesilis* strain IC10 and *S. rochei* strain Y28. Tree was calculated using neighbor-joining method illustrating the taxonomic position of the strains with related species. Accession numbers of the sequences are given in parentheses. The sequences of *Alloactinosynnema album* 03-9939T (EU438907) was used as outgroup. Bootstrap values are based on 1000 resampling and shown at the branching points. Bar indicates 0.01 substitutions per nucleotide position.

Phenotypical characterization was performed using aerial hyphae and spore chains color of 10-days-old bacterial culture on ISP media. On the medium ISP2, isolates TO612 and Y17 were differentiated from IC10, IC13, IT25, and Y28 according to the color of spore chains. On the medium ISP3, isolates Y28, IT25, and IC10 were distinct from the others based on the color of aerial hyphae. Further, on the medium ISP4, isolate IC13 was differentiated from IC10 by its color of aerial hyphae. Also, these two isolates were recognizable on ISP3 according to the color of their aerial hyphae. Isolates IC10 and Y28 were well distinguished from TO612, Y17 and IC13 using ISP media and from IT25 based on melanin production. Isolates IC10 and Y28 slightly were different from each other on ISP2 medium (Supplementary Figure S2). Physiological tests showed only IC13 had potential to grow at 42°C (Table 4). All six isolates were able to grow on NaCl 6 % while only isolates Y28 and Y17 were able to grow on medium containing NaCl 10 % (Table 4). The preliminary phylogenetic analysis of the 16S rRNA gene sequences showed IC10 and Y28 were closely related to species of the genus *Streptomyces*. The phylogenetic tree constructed from 16S rRNA sequences showed that isolates IC10 and Y28 are two members of *Streptomyces* genus with more than 99.5% sequence similarity to *S. enissocaesilis* NRRL B-16365^T^ and *S. rochei* NBRC 12908^T^, respectively (Figure 2).

**Table 4:** Cultural characteristics of superior PGP antagonistic isolates.

Isolate	Color of aerial hyphae – spore chains on ISP media			Melanin production	Growth (in/on)				GeneBank acc. number
	ISP2	ISP3	ISP4	Tyrosine/no tyrosine media	42°C	NaCl 6%	NaCl 10%	NaCl 13%	
IT25	Yellow – Light purple	Colorless – Light purple	Yellow – purple	−/−	−	++	−	−	MG722685
TO612	Yellow – Light greenish	Yellow – Yellow	Yellow – White	+/+	−	+	−	−	MG786938
Y17	Yellow – Greenish	Yellow – Olive	Dark yellow – White	+/+	−	++	+	−	MG786894
Y28	Yellow – light purple	Colorless – Dark purple	Dark yellow – Purple	−/+	−	++	+	−	MG786896
IC10	Purple –Yellow	Colorless – Purple	Yellow –Purple	−/+	−	+	−	−	MG654776
IC13	Yellow – Light purple	Dark yellow – Purple	Yellow – Dark gray	−/−	+	+	−	−	MG676358

### Tomato Growth Promotion Activity of the Superior Isolates

**FIGURE 3:**
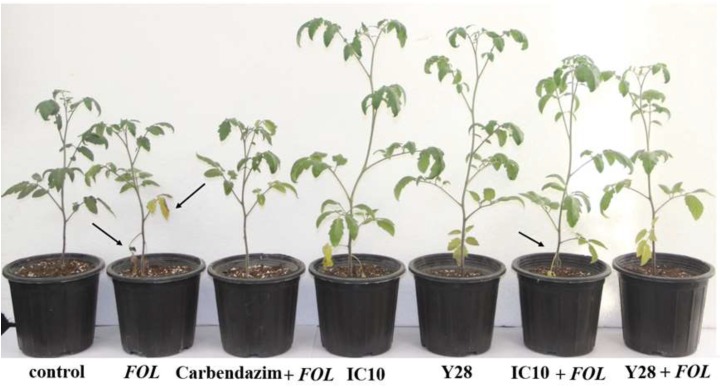
Biocontrol of tomato wilt and stunt caused by *FOL* using *S. enissocaesilis* strain IC10 and *S. rochei* strain Y28 in greenhouse conditions. Data recorded 60 days after bacterial treatment. Stunting is seen in infected plants. Arrows showing wilting symptoms (chlorosis and dried leaves) in *FOL* inoculated plants.

Strains TO612, Y28, IC10, and IC13 significantly increased shoot length compared to control plants. Strains Y28 and IC10 increased shoot length up to 20% compared to control (Figure 3 and Table 5). Strains Y17, Y28, IC10 and IC13 significantly (*p* < 0.05) increased fresh shoot weight while only strains IC10 and IC13 increased shoot dry weight. The increases in the shoot fresh and dry weight were in a range 29–36% and 22–37%, respectively (Table 5). *Streptomyces* strains did not increase root fresh and dry weight while induced root lateral branching (hairy roots) compared to control (data not shown).

**Table 5:** Effect of superior PGP antagonistic isolates (IT25, TO612, Y17, Y28, IC10, and IC13) on tomato growth parameters in greenhouse conditions.

Treatment	Shoot length (cm)	Shoot fresh weight (g)/plant	Shoot dry weight (g)/plant	Root fresh weight (g)/plant	Root dry weight (g)/plant
Negative control	24.18 ± 0.55^c∗^	11.36 ± 0.60^b^	1.79 ± 0.14^b,c^	3.61 ± 0.14^a^	1.72 ± 0.10^a^
IT25	24.87 ± 0.60^c^	10.19 ± 0.35^b,c^	1.39 ± 0.16^c^	2.36 ± 0.35^a,b^	0.90 ± 0.19^d,e^
TO612	29.37 ± 0.32^a^	11.63 ± 0.23^b^	1.87 ± 0.19^b^	2.78 ± 0.17^a,b^	0.81 ± 0.16^d,e^
Y17	26.50 ± 0.20^b,c^	15.47 ± 0.30^a^	2.18 ± 0.10^a,b^	3.61 ± 0.19^a^	1.51 ± 0.25^b^
Y28	29.00 ± 0.56^a,b^	14.97 ± 0.49^a^	2.16 ± 0.15^a,b^	2.88 ± 0.12^a,b^	1.23 ± 0.18^c^
IC10	28.90 ± 0.25^a,b^	15.37 ± 0.38^a^	2.31 ± 0.39^a^	1.64 ± 0.32^b^	0.92 ± 0.12^d,e^
IC13	28.62 ± 0.29^a,b^	14.70 ± 0.65^a^	2.45 ± 0.22^a^	1.78 ± 0.13^b^	0.80 ± 0.24^d,e^

*Data recorded 60 days after bacterial treatment. ^∗^Same letters within each column represent non-significant difference at 5%, according to Duncan’s tests using the GLM procedure (df = 9, F = 7.16, and p < 0.05).*

### Biocontrol Potential of the Superior Isolates

Biocontrol efficacy of the superior isolates against *FOL* causing wilt disease was evaluated in the greenhouse and compared to the chemical fungicide Carbendazim^®^. The high level of disease severity (4.3) was observed in positive control. In Carbendazim^®^ treatment, disease severity was 2.1. Disease severity in the plants treated with IC10 and TO612 were 1.6 and 1.9, respectively. Strain IC10 increased control value by 12.5% compared to the chemical fungicide (Figure 3). Minimum shoot length, fresh and dry weight were related to positive control and minimum fresh and dry root weight were related to Carbendazim^®^. All superior isolates significantly increased shoot length, fresh and dry weight of shoot, compared to positive control (Table 6). Strains IC13 increased tomato total dry weight by 30 and 51% compared to control and Carbendazim^®^ respectively. Strains Y28 and IC10 with the highest PGP and biocontrol activity were selected to determine their role in the induction of tomato-systemic resistance through antioxidant enzymes and defense-related genes.

**Table 6:** Biocontrol of tomato wilt and stunt caused by *F. oxysporum* f.sp. *lycopersici* race 3 (*FOL*) using superior PGP antagonistic isolates (IT25, TO612, Y17, Y28, IC10, and IC13) in greenhouse conditions.

Treatments	Shoot length (cm)	Shoot fresh weight (g)/plant	Shoot dry weight (g)/plant	Root fresh weight (g)/plant	Root dry weight (g)/plant	Disease severity	Control value (%)
Negative control	23.62 ± 0.25^b∗^	11.20 ± 0.63^a,b^	1.35 ± 0.35^a,b^	3.31 ± 0.10^a^	1.41 ± 0.33^a^	1.0^d^	–
Positive control (FOL)	14.00 ± 0.28^d^	5.56 ± 0.34^c^	0.78 ± 0.18^c^	1.66 ± 0.33^a,b^	0.37 ± 0.29^c,d^	4.3^a^	–
Carbendazim^®^+FOL	15.25 ± 0.22^d^	13.00 ± 0.25^a,b^	2.20 ± 0.16^a^	0.23 ± 0.38^b^	0.18 ± 0.14^d^	2.1^a,b,c^	71.9^a,b^
IT25+FOL	20.20 ± 0.43^c^	10.23 ± 0.27^b,c^	1.77 ± 0.55^a,b^	2.48 ± 0.49^a^	1.00 ± 0.37^a,b^	2.3^a,b^	68.8^b,c^
TO612+FOL	19.00 ± 0.20^c^	12.77 ± 0.32^a,b^	2.40 ± 0.38^a^	2.90 ± 0.20^a^	1.40 ± 0.38^a^	1.9^c,d^	78.1^a^
Y17+FOL	19.50 ± 0.35^c^	13.38 ± 0.26^a,b^	2.09 ± 0.58^a^	2.76 ± 0.37^a^	0.94 ± 0.29^a,b^	2.0^b,c^	75.0^b,c^
Y28+FOL	26.70 ± 0.15^a^	11.88 ± 0.75^a,b^	2.02 ± 0.60^a^	2.94 ± 0.28^a^	0.59 ± 0.33^b,c^	2.0^b,c^	75.0^b,c^
IC10+FOL	26.60 ± 0.35^a^	12.83 ± 0.10^a,b^	2.07 ± 0.38^a^	1.83 ± 0.18^a,b^	0.96 ± 0.24^a,b^	1.6^c,d^	84.4^a^
IC13+FOL	21.33 ± 0.39^c^	15.00 ± 0.29^a^	2.45 ± 0.41^a^	2.54 ± 0.30^a^	1.15 ± 0.31^a,b^	2.1^a,b,c^	71.9^a,b^

*Data recorded 60 days after bacterial treatment. Disease severity was assessed on a scale of 1 to 5 (1, symptoms free; 5, severe symptoms). ^∗^Same letters within each column represent non-significant difference at 5%, according to Duncan’s tests using the GLM procedure (df = 11, F = 3.63, and p < 0.05).*

### Antioxidant Enzymes Activity

**FIGURE 4:**
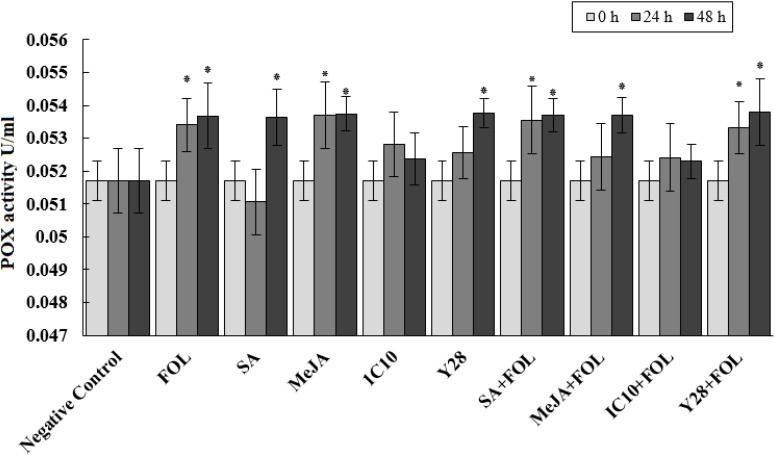
Effect of biological (*S. enissocaesilis* strain IC10 and *S. rochei* strain Y28) and chemical (SA and MeJA) treatments on the induction of peroxidase (POX) activity in tomato leaves non-inoculated (left) and inoculated (right) with *FOL* at different time intervals of inoculation (*df* = 29; *F* = 2.90; *P* < 0.01). Data represent the mean values ± SE of three biological replicates. Negative control: untreated and non-inoculated. In each treatment, the values marked with an asterisk are significantly (*P* < 0.05) different from negative control at time point 0.

**FIGURE 5:**
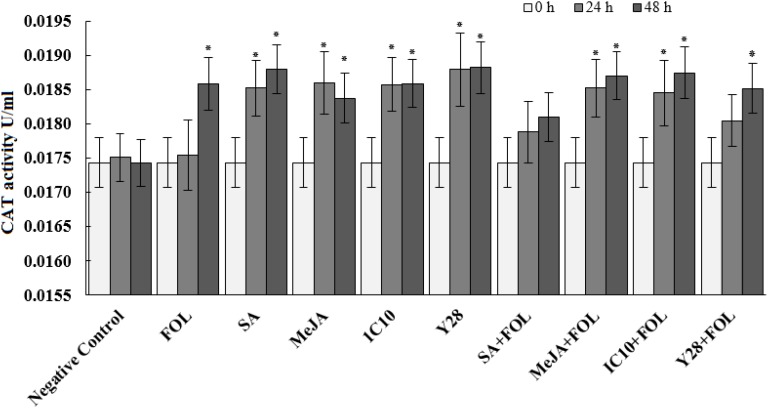
Effect of biological (*S. enissocaesilis* strain IC10 and *S. rochei* strain Y28) and chemical (SA and MeJA) treatments on the induction of catalase (CAT) activity in tomato leaves non-inoculated (left) and inoculated (right) with *FOL* at different time intervals of inoculation (*df* = 29; *F* = 30.73; *P* < 0.01). Data represent the mean values ± SE of three biological replicates. Negative control: untreated and non-inoculated. In each treatment, the values marked with an asterisk are significantly (*P* < 0.05) different from negative control at time point 0.

Peroxidase activity significantly increased 24 h after inoculation of plants with *FOL*. Further, MeJA individually increased the enzyme activity at the same time interval. Plants inoculated with *FOL* in SA or Y28 treatment increased POX activity after 24 h like positive control (*FOL*). In SA treatment, POX activity increased 48 h after exogenous application. The level of POX activity remained constant in plant treated with *FOL* and MeJA and also SA and Y28 inoculated with *FOL*. In soil treated with strain IC10 and inoculated or non-inoculated with the pathogen, POX activity did not increase during the experiment time (48 h) (Figure 4). CAT activity of tomato plants increase 48 h after *FOL* inoculation. Treatments including bacterial strains and plant hormones increased CAT activity after 24 h. Pathogen inoculation slightly suppressed CAT activity in the SA treatment (Figure 5).

### Quantitative Real-Time PCR Analysis of the Defense-Related Genes

**FIGURE 6:**
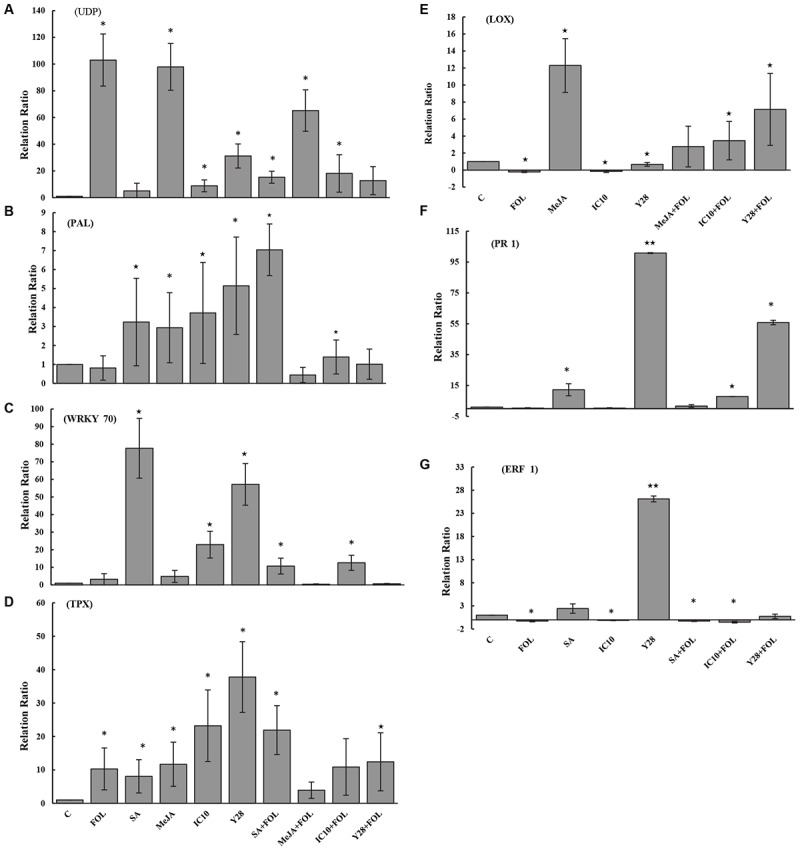
The relative level of gene expression (fold) determined by qRT-PCR of seven target genes including *UDP* **(A)**, *PAL* **(B)**, *WRKY70* **(C)**, *TPX* **(D)**, *LOX* **(E)**, *PR1* **(F)**, and *ERF1* **(G)** versus reference control (*Tubulin* gene) in tomato “Riogrande” treated with chemical (MeJA and SA) or biological agents (*S. enissocaesilis* strain IC10 and *S. rochei* strain Y28) 48 h after inoculation with *FO*L/ distilled water. Untreated and non-inoculated plants were considered as control (C) and as reference sample. Standard error represents for three biological replicates. Positive values of fold change indicate up-regulation while negative values indicate down-regulation. The values marked with one and two asterisk are significantly different from control at *P* < 0.05 and *P* < 0.01, respectively.

Plant inoculation with *FOL* and MeJA increased UDP-glycosyltransferase (*UDP*) transcripts by 103- and 98-fold, respectively, compared to control. Although *Streptomyces* strains induced *UDP* transcription, they significantly suppressed gene expression in the plants inoculated with *FOL* (Figure 6A). Treatments including *Streptomyces* strains and plant hormones and not *FOL* significantly increased phenylalanine ammonia-lyase (*PAL*) transcripts compared to control. Salicylic acid and strain IC10 also retained their induction effect in plants inoculated with *FOL* (Figure 6B). The results revealed that plants treated with SA, IC10, Y28, SA-*FOL*, and IC10-*FOL* significantly stimulated (up-regulated) *WRKY70* expression. Treatments of *FOL* and MeJA did not increase relative expression of *WRKY70* in tomato plants (Figure 6C). Tomato peroxidase (*TPX1*) transcripts was significantly up-regulated in all treatments including *Streptomyces* strains and plant hormones. Salicylic acid and strain Y28 also induced *TPX1* gene expression in plants inoculated with *FOL* (Figure 6D). The highest lipoxygenase (*LOX*) gene expression level (38- fold) was detected in Y28. There was a significant down-regulation of *LOX* gene in *FOL* and IC10 treatments. On the contrary, MeJA and Y28 stimulated *LOX* expression. Induced *LOX* expression was also observed in plants treated with bacteria and inoculated with *FOL* (Figure 6E). In a similar pattern, SA and Y28 significantly increased pathogenesis related protein 1 (*PR1*) and ethylene response factor 1 (*ERF1*) transcripts. Strains IC10 and Y28 also were able to stimulate the *PR1* expression in the presence of *FOL* (Figure 6F,G).

### Correlation Analysis

There was a significant relationship between siderophore and IAA production (*r* = 0.36). Besides, a correlation was found between growth inhibition and protease activity (*r* = 0.31). Moreover, growth inhibition of *FOL* in dual culture assay was significantly correlated to siderophore production (*r* = 0.64) (Table 7). A significant negative correlation (−0.63) was observed between growth inhibition (*in vitro* assay) and disease severity (*in vivo* assay).

**Table 7:** Pearson correlation coefficients (r) between PGP traits (*in vitro* assays) and biocontrol activity (*in vivo* assay).

Pearson correlation	*In vitro* assays		*In vivo* assay
Variables	IAA production	Fungal growth inhibition (%)	Disease severity
Protease activity	0.44^ns^	0.31^∗^	−0.47^ns^
Siderophore production	0.36^∗^	0.64^∗∗^	0.24^ns^
IAA production	1	0.47^∗^	−0.01^ns^
Fungal growth inhibition			−0.63^∗^

*ns, non-significant; ^∗^P < 0.05; and ^∗∗^P < 0.01.*

## Discussion

Almost half of tomato and cucumber greenhouses in Iran (more than 7000 hectares) is located in Yazd, Isfahan, and Kerman provinces. Amount and type of organic fertilizer and soil used to prepare the greenhouse growth bed are different in these provinces. Farmers in Yazd and Kerman use sand from the bed of the nearest rivers to their greenhouse and farmers in Isfahan use soil of the greenhouse floor as a raw material to prepare culture bed (private conversation). Our results showed distribution of the bacterial isolates with the *Streptomyces* appearance in the three provinces is slightly different. Among the isolates that showed a PGP trait, a relatively stable share (42–46%) belonged to Isfahan. The contribution of the *Streptomyces* isolated from Yazd or Kerman in each PGP trait was not always constant showing *Streptomyces* with several PGP traits in the field soil is greater than that of rivers sand. A previous study on *Streptomyces* showed that bacterial diversity was more affected by the habitat than the population (Davelos et al., 2004). The restriction of some nutrients in soil may be a major contributor to the change in the microbial diversity due to natural selection.

Growth ability on a nitrogen-free medium and IAA and siderophore production were three dominant characteristics of *Streptomyces* isolated from vegetable rhizosphere soils. A lower percentage (30%) of the isolates had the potential for solubilizing phosphate. Besides, the location of isolation was also effective on this ratio. There was a significant positive correlation between IAA and siderophore production, which may remind a complementary role of the siderophore in plant growth promotion activity (i.e., in addition to its role in antagonistic activity). Such correlation was not observed between other traits involved in antagonist activity and IAA production.

The ability of *FOL* growth inhibition was very different between *Streptomyces* isolates showing PGP properties. Isolate CU122 with four PGP traits, was not able to inhibit *FOL* growth. Failure to inhibit the pathogen growth was correlated to the inability of isolate CU122 to produce chitinase and protease enzymes. On the contrary, isolate IC10 with same *in vitro* PGP features as well as enzymes activity inhibited *FOL* growth very well. The correlation of protease and chitinase activity with inhibition of *FOL* growth was positive and respectively, quantitative and non-quantitative. Only two exceptions did not adhere to this relationship. Isolates TO612 and KH12, which did not show enzyme activity, inhibited *FOL* growth in an acceptable range (equal to or greater than 30%).

This finding revealed that although chitinolytic activity of *Streptomyces* and digestion of the fungal cell wall is an effective mechanism of growth inhibition (Gherbawy et al., 2012), other mechanisms such as antibiosis and even an enzyme like protease (Xu et al., 2016) that is less studied are also involved in antagonism. Interestingly, there was a significant correlation between IAA and siderophore productions and *FOL* growth inhibition, showing relationship between PGP features and antagonistic activities in *Streptomyces*. IAA production is a common characteristic among antagonistic species of *Streptomyces* (Sreevidya et al., 2016). It seems that IAA production alone does not cause plant growth, and other traits, such as siderophore production, also has an important role in increasing plant growth. In this regard, Kunova et al. (2016) showed that the antagonistic isolates producing IAA did not increase growth of different vegetable species. These isolates were not able to produce siderophore. The significant negative correlation that was observed between *FOL* growth inhibition (*in vitro* assay) and disease severity (*in vivo* assay) confirmed a need for *in vitro* tests to select the best biocontrol strains. Recently, Wu et al. (2019) reported that a decreased percentage disease index of rice sheath blight caused by *Rhizoctonia solani* was associated with an increase in *Streptomyces* derived antifungal agent concentration. They concluded that the antifungal agent reduces sheath blight symptoms via exerting a strong antagonistic activity against *R. solani* both *in vivo* and *in vitro* conditions. *S. rochei* ACTA1551 from Greek (Kanini et al., 2013), *S. miharaensis* KPE62302H (Kim et al., 2012), and *S. psammoticus* KP1404 (Kim et al., 2011) from Korea, *S. plicatus* from Egypt (Abd-Allah, 2001) and *S. griseorubens* E44G from Saudi Arabia (Rashad et al., 2017) were reported as successful biocontrol agents to control tomato *Fusarium* wilt. In the previous reports, the pathogen race has not been determined and to our knowledge, this is the first report of biocontrol of Fusarium wilt caused by *F. oxysporum* Schlecht. f. sp. *lycopersici* (Sacc.) race 3 in tomato with *Streptomyces* strains.

Strain Y28 and IC10 were close to *S. rochei* and *S. enissocaesilis*, respectively and are grouped in a clade on the phylogenetic tree. These PGP strains were isolated from the rhizosphere soils of two greenhouses in two different locations with a distance of 300 kilometers. The high similarity in PGP, and biocontrol activity of these two strains revealed that evolutionary process can keep PGP traits and biological control activities together.

All plants treated with SA, MeJA and Y28 or inoculated with *FOL* increased POX activity during 48 h after *FOL* inoculation. The POX activity in hormone or PGPR treated plants was not affected by *FOL* inoculation. Despite the similar biocontrol activity, isolates IC10 and Y28 had a different effect on the induction of plant POX activity. None of the plants treated with IC10, inoculated or non-inoculated with *FOL*, increased POX activity. Peroxidase is considered an important pathogen-related protein (PR-protein) or defense protein involved in many physiological responses of plants to biotic stresses. Contribution to biosynthesis of lignin (Chittoor et al., 1997) and antimicrobial compounds such as phytoalexins and quinones (Mayer, 2006) are two well-known POX roles associated with ISR. Increased peroxidase activity of cucumber was reported in plants showing Fusarium wilt (Zhao et al., 2012) and also plant treated with *Streptomyces* as biocontrol agent (Salla et al., 2016).

Peroxidase, as well as CAT, are involved in plant antioxidant defense system and reduce the harmful effects of stresses by scavenging of ROS (Das and Roychoudhury, 2014). In this study, the effects of *FOL*, plant hormones and PGP strains to induce CAT activity were the same. Induction of systemic resistance through defense-related enzymes POX and CAT in tomato plants treated with salicylic acid or *Pseudomonas fluorescens* was reported by Nikoo et al. (2014). They showed that pathogen inoculation of plants treated with bacterial or hormonal elicitors increased both POX and CAT activity in higher levels compared to non-inoculated treated plants or plants only inoculated with pathogen. This association was not observed in our experiment.

Relative expression of several candidate genes encoding transcription factors (*WRKY70* and *ERF1*), *PR1*, *TPX1*, *UDP*, *LOX*, and *PAL* was evaluated in this study. The plant-specific transcription factor WRKY70 is an important factor in *Arabidopsis* signaling pathways and its expression is activated by SA and repressed by JA. Ren et al. (2008) showed that overexpression of *WRKY70* reduced JA responses and mutually JA treatment inhibited *WRKY70* expression. On the contrary, WRKY70 is known as a positive regulator of SA-mediated defense because increases *PR1* which is often studied as a marker gene for SA-dependent defense signaling (Li et al., 2004). It is reported that PGP *Bacillus cereus* AR156 stimulated the transcription of *WRKY70* in *Arabidopsis* leaves. According to their study, *WRKY70* modulated *B. cereus*-triggered ISR through activating SA signaling pathway (Wang et al., 2018). Our results are in accordance with the reported studies and showed induction of *WRKY70* transcription upon treatment with SA, and not with MeJA, in *FOL* inoculated and non-inoculated plants. IC10 and Y28 also significantly induced transcription of *WRKY70* and IC10 continued to induce gene expression after *FOL* inoculation. Following increased *WRKY70* transcripts, expression of *PR1* increased in SA and Y28 treatments. Great increase in *PR1* transcript abundance by Y28 and not IC10 indicates that these two PGPRs stimulate different pathways to elicit defense priming in tomato plant.

UDP-glucose dependent hydroquinone: O-glucosyltransferase (arbutin synthase) is a member of glycosyltransferases catalyze production of arbutin in higher plants (Arend et al., 2000a). Arbutin is a phenolic glycoside that has antimicrobial and antifungal activity (Kundakovic et al., 2014). Arbutin synthase is a multifunctional enzyme converting various natural products, xenobiotics and toxins (Arend et al., 2000b). There is no report about induction of *UDP* in response to *F. oxysporum* or *Streptomyces* PGPRs in tomato plant. A previous report revealed that induction of members of *UDP* family, *UGT73B3* and *UGT73B5* is necessary during the hypersensitive response of *Arabidopsis* to plant pathogen *Pseudomonas syringae* (Langlois-Meurinne et al., 2005). Also treatments of *Arabidopsis* with SA and MeJA induced the expression of another gene family member *UGT73C5* involved in mycotoxin detoxification (Poppenberger et al., 2003). Increased *UDP* transcript in *FOL* inoculated tomato can be attributed to the role of arbutin in detoxification of fungal toxin or its antimicrobial effect (Kuzniak et al., 2015). The increased expression of this gene in treatment of MeJA and not SA represents its role in ISR. Compared to *FOL* inoculation, a significant lower level of *UDP* expression was observed in plants treated with IC10 and Y28 highlighting the potential role of these PGP strains in tomato defense priming.

*FOL*, SA, MeJA and PGPRs induced expression of *TPX1*. *TPX1*, peroxidase encoding gene, is involved in the synthesis of lignin and suberin (Quiroga et al., 2000). Here, *TPX1* expression induction by Y28 and IC10 revealed their role in ISR. *TPX1* expression at the lower level was observed 48 h after *FOL* inoculation. Different POX activities in plants treated with PGPRs could be related to the *TPX1* transcript abundance.

Phenylalanine ammonia lyase encoded by *PAL* is involved in the biosynthesis of salicylic acid and defense compounds including flavonoids, phenylpropanoids and lignin. Induction of PAL activity in response to various stimulants such as tissue plant pathogens and hormones was reported (Edwards et al., 1985). In this study, induced expression of *PAL* in bacterial and hormonal treatments was observed and there was no difference between the induction effects of bacterial strains. The induction of PAL enzymatic activity and *PAL* gene expression in tomato plants upon pre-treatment with *Bacillus thuringiensis* (Akram et al., 2013) and the mycorrhizal fungus *Funneliformis mosseae* (Song et al., 2015) were also reported.

Members of the ERF protein family are transcription factors involved in ET/JA or SA signaling pathways and cause moderate disease resistance responses in various plant species (Liang et al., 2008). Induced expression of *ERF1* in *Arabidopsis* plants treated with a PGP biocontrol strain of *Paraburkholderia phytofirmans* in response to a model plant pathogen *P. syringae* pv. tomato DC3000 was reported recently (Timmermann et al., 2017). Likewise, induction of *ERF1* by treatment of Y28 tomato was observed in the present study too. On the contrary, plants treated with IC10 decreased *ERF1* expression. Our data supports the hypothesis that studied PGP strains regulate tomato defense responses through different molecular pathways possibly acting at transcriptional level.

*LOX* is a signaling molecule involved in pathogen plant resistance. Strain Y28 slightly induced *LOX* expression in non-pathogen inoculated plants. *FOL* inoculation and IC10 treatment caused a significant decreased in transcript level of *LOX* compared to the control plants. *FOL* inoculation of both bacterial treatments caused an induction of gene expression. Our finding is consistent with Mariutto et al. (2011) that showed tomato plants treated with PGP *Pseudomonas putida* did not induce *LOX* transcription before inoculation with the pathogen *B. cinerea.* Although transcripts of *LOX* for both strains were higher in *FOL* inoculated plants, gene expression levels in non-inoculated bacterial treatments were different significantly.

## Conclusion

The superior PGP antagonistic *Streptomyces* strains, show biocontrol activities against Fusarium wilt of tomato caused by *F. oxysporum* Schlecht. f. sp. *lycopersici* (Sacc.) race 3. Significant positive correlation between siderophore and IAA production and siderophore accumulation and growth inhibition showed a relationship between PGP and antagonistic traits. A significant negative correlation between growth inhibition (*in vitro* assay) and disease severity (*in vivo* assay) confirmed a need for *in vitro* tests to select the best biocontrol strains. The correlation of PGP traits with biocontrol activity in phylogenetically close species isolated from distant habitats refers to the role of the natural selection in preserving traits that give superiority to bacteria in the rhizosphere. Here we showed biocontrol *Streptomyces* stimulate plant defense system through different molecular pathways at the transcriptional level.

## Author Contributions

NS and AS designed and directed the research. MS gave advice during experiments. SA carried out all experiments. All authors contributed to the interpretation of the results. SA wrote the manuscript with help from NS and AS. All authors read and approved the final manuscript.

## Conflict of Interest Statement

The authors declare that the research was conducted in the absence of any commercial or financial relationships that could be construed as a potential conflict of interest.

## Supplementary Material

FIGURE S1 Identification of *Fusarium oxysporum* f. sp. *lycopersici* (*FOL*) physiological race 3 by selective primers. Polymerase chain reactions carried out using unif, sp13, sp23, and sprl primer sets. 1 kb: I kb ladder; C, negative control.

FIGURE S2 Bacterial colonies, *S. enissocaesilis* strain IC10 and *S. rochei* strain Y28, on ISP2 medium 10 days after cultivation.
