# Supplementary figures and images for: Streptomyces Strains Induce Resistance to Fusarium oxysporum f. sp. lycopersici Race 3 in Tomato Through Different Molecular Mechanisms
Source: Front Microbiol. 2019 Jul 3;10:1505. doi: 10.3389/fmicb.2019.01505 (PMC6616268; doi:10.3389/fmicb.2019.01505)

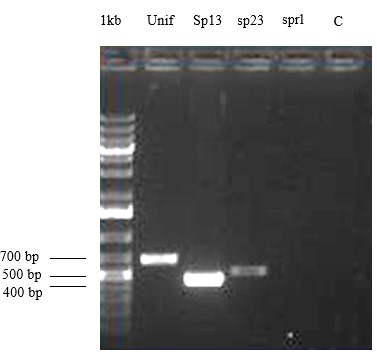

Supplement: FIGURE S1 — Identification of Fusarium oxysporum f. sp. lycopersici (FOL) physiological race 3 by selective primers. Polymerase chain reactions carried out using unif, sp13, sp23, and sprl primer sets. 1 kb: I kb ladder; C, negative control. [file Image_1.jpg]

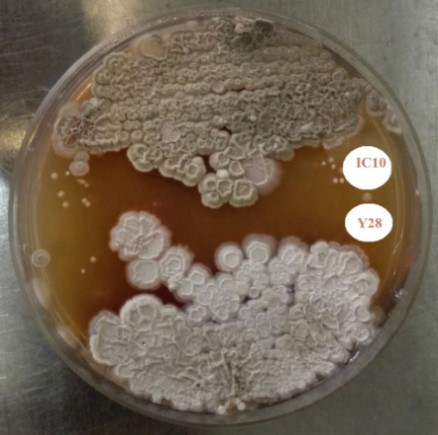

Supplement: FIGURE S2 — Bacterial colonies, S. enissocaesilis strain IC10 and S. rochei strain Y28, on ISP2 medium 10 days after cultivation. [file Image_2.jpg]
